# Authors’ response: Highly pathogenic influenza A(H5N1) viruses in farmed mink outbreak contain a disrupted second sialic acid binding site in neuraminidase, similar to human influenza A viruses

**DOI:** 10.2807/1560-7917.ES.2023.28.7.2300109

**Published:** 2023-02-16

**Authors:** Montserrat Agüero, Isabella Monne, Azucena Sánchez, Bianca Zecchin, Alice Fusaro, María José Ruano, Manuel del Valle Arrojo, Ricardo Fernández-Antonio, Antonio Manuel Souto, Pedro Tordable, Julio Cañás, Francesco Bonfante, Edoardo Giussani, Calogero Terregino, Jesús Javier Orejas

**Affiliations:** 1 Laboratorio Central de Veterinaria (LCV), Ministry of Agriculture, Fisheries and Food, Algete, Madrid, Spain; 2 Istituto Zooprofilattico Sperimentale delle Venezie (IZSVe), Legnaro, Italy; 3 Counselling of Health, Xunta de Galicia, A Coruña, Spain; 4 Galician Mink Breeders Association, Santiago de Compostela, A Coruña, Spain; 5 Livestock Service, Counselling of Rural Affairs, Xunta de Galicia, A Coruña, Spain; 6 Animal Health Service, Counselling of Rural Affairs, Xunta de Galicia, A Coruña, Spain; * These authors contributed equally to this work and share first authorship.

**Keywords:** mink, Influenza A, H5N1, highly pathogenic avian influenza, Spain

**To the editor:** We would like to thank the authors of the letter for their valuable contributions to our article describing a highly pathogenic avian influenza (HPAI) virus outbreak in farmed minks in Spain in October 2022 [1].

de Vries et al. highlighted that the HPAI A(H5N1) viruses identified in Spain in minks contain a disrupted second sialic acid binding site (2SBS) in neuraminidase, similar to human influenza A viruses [2,3]. In addition, they also pointed out that the mutations at position S369I and I396M of neuraminidase (NA) implicated in the loss of the 2SBS had already been acquired in avian hosts before the emergence of the outbreak in minks. We agree with the authors that the evolution of NA in avian viruses in relation to altered receptor-binding properties is an aspect that should not be overlooked. Based on the currently available genetic data, at the time of writing of the article, among the HPAI H5 viruses sequenced in Europe since autumn 2020, mutations S369I and I396M of the NA had already been identified in combination in six H5N1 viruses identified in gulls in the Netherlands and Belgium between June and October 2022. In particular, in Europe, these mutations were detected only in viruses belonging to the A/gull/France/22P015977/2022-like genotype [1]. This genotype emerged from reassortment events with viruses of the gull-adapted A(H13) subtype from which it acquired the NP, PA and NS genes [4]. Since its first identification in May 2022, the genotype has been extensively detected in colony-breeding seabird species, mainly European herring gulls (83.3% of the available sequences of this genotype from birds are seabird species). What has caused these two mutations to appear in the generally conserved 2SBS in NA of this genotype is a matter of speculation. Considering that the H13 and H16 subtype viruses, which are particularly adapted to gulls, differ from avian viruses of other species by their receptor-binding properties [5], it cannot be excluded that the NA mutations might be related to adaptation of these viruses to this specific avian host. However, a founder effect also needs to be considered. Whatever the origin of these mutations might be, additional in vitro and in vivo studies are needed to evaluate the biological impact of the 2SBS disruption in the specific genome constellation of the A/gull/France/22P015977/2022-like genotype, identified in both the avian and mammalian hosts.

This applies also to other mutations shown to alter the receptor binding specificity in vitro, and which have been described in viruses of clade 2.3.4.4b A(H5) identified in the avian host in Europe since 2020 [6]. These have not been described in our rapid communication, as our focus was to highlight the mutations detected in the viruses identified in minks in Spain compared with the most closely related H5N1 viruses characterised in the avian population. Their actual effect on the biological characteristics of these circulating viruses still remains unexplored.

The sequence data available indicate that the A/gull/France/22P015977/2022-like genotype is not prevalent in Europe and represents 9.1% of all the sequences available since its emergence in May 2022 (up to 10 February 2023). However, bias exists between publicly accessible sequences and the number of outbreaks reported in the avian and mammal populations. The mutations described by de Vries remind us about the importance of carefully analysing whole genome sequences and vigilantly monitoring virus evolution in each host, whether domestic or wild. Any country that notifies an outbreak should ideally have access to sequencing and data sharing mechanisms. Genomic surveillance is crucial to quickly identify any virus with zoonotic potential that could pose a threat to animal and public health.
